# Development and validation of a dynamic light scattering-based method for viral quantification: A straightforward protocol for a demanding task

**DOI:** 10.1371/journal.pone.0324298

**Published:** 2025-05-27

**Authors:** Rene A. Navarro-Lopez, Erika Silva-Campa, Gerardo Santos-López, Adriana Garibay-Escobar

**Affiliations:** 1 Departamento de Investigación en Física, Universidad de Sonora, Hermosillo, Sonora, México; 2 Posgrado en Ciencias de la Salud, Departamento de Ciencias Químico-Biológicas, Universidad de Sonora, Hermosillo, Sonora, México; 3 Centro de Investigación Biomédica de Oriente, HGZ No. 5, Instituto Mexicano del Seguro Social, Metepec, Puebla, México; 4 Departamento de Ciencias Químico-Biológicas, Universidad de Sonora, Hermosillo, Sonora, México; Instituto Butantan, BRAZIL

## Abstract

This study aimed to develop a reliable, rapid method for viral particle quantification using Dynamic Light Scattering (DLS) as an alternative to traditional virological techniques. Conventional methods, such as plaque assays, though widely recognized, are time-intensive and depend on the observation of cytopathic effects, which can be subjective. In this study, Influenza A virus (IAV) supernatants were quantified using DLS and subsequently compared with titers determined by plaque assays and TCID_50_. DLS measures the fluctuations in light scattering caused by particles in Brownian motion, allowing direct, non-destructive measurement of viral concentration within minutes. Results indicated a strong correlation between the DLS-derived viral titers and those obtained from plaque assays (R^2^ = 0.9967) and TCID_50_ (R^2^ = 0.9984), demonstrating DLS potential as a complementary or alternative method for rapid quantification. The technique non-reliance on cell viability and ability to measure intact viral particles enhance its applicability for assays where infectivity is not the primary concern. Additionally, DLS facilitated the detection of complete viral particles, which is advantageous for vaccine development and antiviral testing. The protocol’s reproducibility across various dilutions, coupled with minimal sample preparation requirements, underscores DLS as a feasible quantification method adaptable for different viral strains. Limitations, including the inability to distinguish between infectious and non-infectious particles, suggest that while DLS serves as a valuable initial quantification tool, further infectivity assessments may be required for comprehensive viral characterization.

## Introduction

Accurate quantification of infectious virus particles is fundamental to virological research. Traditionally, viral titers have been determined using methods such as plaque assay (PA) [1] and 50% Tissue Culture Infectious Dose (TCID_50_) [2]. These methods rely on the observation of cytopathic effects in cell cultures following serial dilutions of virus-containing samples [3]. Also, such traditional viral titration methods are labor-intensive, time-consuming, often requiring several days to complete, depending on viral growth kinetics [4,5]; and prone to subjective interpretation, as their reliance on visual observation of cytopathic effects can introduce variability due to operator-dependent interpretation and potential cell culture artifacts [4,6].

Dynamic Light Scattering (DLS) is a non-destructive analytical technique widely used in nanotechnology, biophysics and materials research to characterize the size and distribution of particles in suspension or solution [7,8].

DLS is based on the principle of Mie scattering, where particles scatter light when illuminated by a monochromatic laser source. Since smaller particles move more rapidly due to Brownian motion and larger particles move more slowly, the fluctuation in the intensity of scattered light over time is directly related to particle size [7,9]. This relationship is mathematically described by the Stokes-Einstein equation [7,8] (**Equation 1**), which allows the calculation of the hydrodynamic radius (R_H_) of the particles:


$$D=\frac{I_{A}-19.69}{1.098\times 10^{-6}}\times dilution$$ (1)

**Equation 1. Stokes-Einstein equation.** D is the translational diffusion coefficient (i.e., particle velocity, m^2^/s), k_B_ is the Boltzmann constant, T is the temperature in Kelvin, η is the viscosity, and R_H_ is the hydrodynamic radius of the particles in meters.

**Fig 1:**
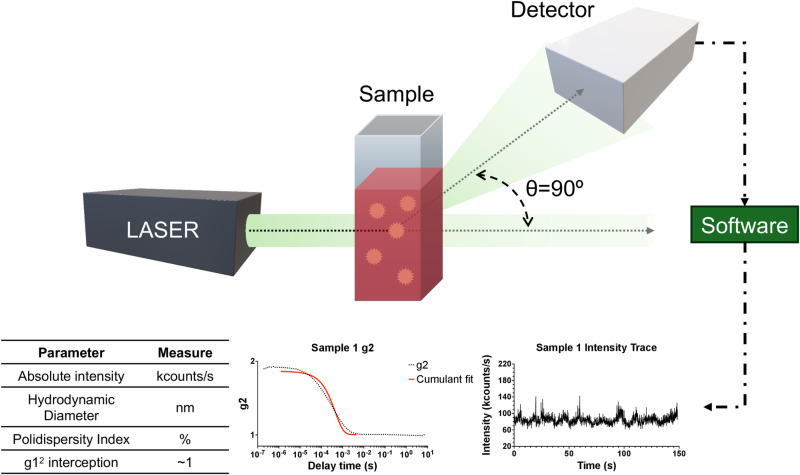
Simplified diagram of a DLS instrument. The sample is contained within a cuvette and is illuminated by a coherent laser beam. The light scattered by the particles in the sample at various angles is detected at 90º by a detector. This scattered light undergoes fluctuations due to the Brownian motion of the particles. By analyzing the correlation of these fluctuations over time, the particle size distribution can be determined.

For DLS measurements, a laser illuminates the sample, and the scattered light is collected at a specific scattering angle (θ) (Fig 1). The detected intensity fluctuations are analyzed using an autocorrelation function, which provides information on the diffusion coefficient and subsequently the particle size distribution [7,8]. The selection of the scattering angle is crucial, as smaller angles (~30°) are more sensitive to larger particles, while wider angles (~90° or higher) provide better resolution for smaller particles.

DLS has a wide range of applications in various fields, including biology, where it is adapted for analyzing the size and structure of biomolecules such as proteins, exosomes, liposomes and viruses [7,8,10]; or for quality control of pharmaceuticals and the development of new drug delivery systems [9,11]. As a non-destructive technique based on light interaction, working conditions can be carefully controlled to avoid sample modification or denaturation, allowing for sample reuse in other assays, even for culture if sterile conditions are sought. Furthermore, the technique’s speed and simplicity allow for rapid and straightforward measurements, without compromising sensitivity, detecting particles from 0.3 nm to 10 μm [7,12].

While DLS is a powerful tool, its suitability depends on several factors. The Polydispersity Index (PdI) is a key parameter in DLS that quantifies the uniformity of particle size distribution. However, when analyzing viruses derived from biological samples, variations in PdI are expected due to inherent biological (aggregation, interactions with host-derived components) and methodological factors (incomplete purification, residual cellular debris, buffer composition and ionic strength). As the technique DLS is most accurate when analyzing monodisperse samples, is important to ensure a PdI below 30% (0.3) keeping on the recommended values for reliable size estimations, as higher values indicate greater heterogeneity, which may affect measurement accuracy [7].

Additionally, sample concentration plays a key role, as excessively high concentrations may lead to multiple scattering events that distort results, while excessive dilution can alter particle behavior. The accuracy of size estimation also depends on the viscosity of the medium, which is a critical parameter in the Stokes-Einstein equation; thus, careful measurement and control of viscosity are essential, particularly in biological samples [9]. Moreover, DLS assumes that particles are spherical when calculating size distributions, meaning that non-spherical, agglomerations or filamentous particles, a behavior already described for some enveloped viruses [13–15], may cause misleading results by affecting the intensity trace profile and the correlation function fit, essential parameters for assessing the quality and reliability of the measurements [11]. The intensity trace profile provides real-time information on the stability of the sample, helping to identify fluctuations caused by aggregation, sedimentation, or contamination that could distort particle size estimation. A stable intensity trace indicates consistent scattering, whereas erratic fluctuations may suggest sample instability or polydispersity. The correlation function fit, on the other hand, reflects how well the mathematical model describes the decay of light scattering intensity over time, which is directly related to particle diffusion and size distribution. A high-quality fit ensures accurate determination of the hydrodynamic diameter, while a poor fit may indicate multimodal distributions, aggregation, or excessive noise in the data. Proper analysis of these parameters is crucial for ensuring reliable data interpretation and assessing the suitability of DLS for a given experimental setup. However, certain limitations can often be mitigated by optimizing the particle concentration [7,16].

Viral quantification is a fundamental tool in virology for understanding viral replication and pathogenesis [2,5,17]. Quantifying viruses in *in vitro* or *in vivo* infection processes helps to study the viral replication cycle, virion production, and disease progression. Additionally, in vaccine or antiviral studies, monitoring viral load before and after treatment allows for the evaluation of their efficacy [3,9,18]. Even in measuring the viral load in patients with chronic viral diseases, it is essential for assessing the effectiveness of therapies and monitoring the prevalence and incidence of viral diseases [17,19].

Viral quantification techniques are based on the detection and measurement of viral components, such as complete virions, viral proteins, viral RNA, or DNA [1,2,6,17]. These techniques can be classified into two main categories: those based on the detection of viral activity and those based on the detection of viral particles or components. Essentially, the difference between the two groups is that when performing a viral titration, what is determined is the minimum concentration of a virus at which activity is still observed (e.g., infected cells with cytopathic effects), while in quantification, the number of particles within a given population is determined (whether infectious or not). The choice of viral quantification technique depends on the specific virus, the availability of resources, and the objectives of the study. Knowing the nature of the sample, the chosen technique must have sufficient sensitivity to detect the expected virus concentrations and sufficient specificity to distinguish the virus of interest from other components that may contaminate the sample matrix, making it essential to include positive and negative controls to ensure the validity of the results.

Other points to consider are that the reproducibility of titrations will depend on experimental experience, as the techniques are laborious, time-consuming, and costly, requiring adequate facilities, materials, and cell culture equipment; not to mention the intrinsic factors of the virus, such as its cultivability, the availability of susceptible cells, and in some cases, the need for cofactors or special supplements for the particular virus.

Currently, several methods for quantifying viral particles have been reported, such as electron microscopy [20], flow cytometry [5,21], interferometry [22], qPCR [17,18], ELISA [18,22], among others; however, these are destructive or require special reagents. Previously, the use of the DLS technique for the characterization of the size and distribution of viral particles has been reported [23–25]; however, in this study, we provide a comprehensive description step by step of a protocol for quantifying viral particles via DLS, addressing best practices and common challenges encountered during its implementation.

## Materials and methods

### Propagation of influenza A virus

To replicate the Influenza A/PR/8/34 H1N1 virus (IAV), MDCK cells were cultured under conditions recommended by the ATCC and infected as previously described [26]. Cells were grown in T25 flasks with 5 mL of Dulbecco’s Modified Eagle’s Medium (DMEM) high glucose (12800, Gibco) supplemented with 5% FBS and 10 IU/mL antibiotics (penicillin-streptomycin, 15140122, Gibco), and incubated at 37 °C in a 5% CO_2_ atmosphere, under this conditions the pH is buffered at 7.4. Once cells reached approximately 80% confluence, the culture medium was removed, cells were washed twice with 1x PBS pH 7.4, 500 µL of viral supernatant in DMEM supplemented with Trypsin-TPCK [2 μg/mL TPCK (T1426, Sigma-Aldrich), Trypsin-EDTA (0.02%-0.1 mM, T2601, Sigma-Aldrich)] was added, and then incubated for 15 min at 37 °C in a 5% CO_2_ atmosphere. Subsequently, 5 mL of DMEM without FBS was added and cultured under the same conditions described above. Simultaneously, a control culture was processed identically, omitting the viral addition. This culture, lacking viral exposure, is termed a mock. At 72 h post-infection (h.p.i.), supernatants were collected.

### IAV supernatants clarification

At 72 h.p.i., IAV-infected cell culture supernatants were collected and clarified by centrifugation to remove cellular debris and particulate material. The samples were centrifuged at 6,000 × g at 4 °C for 30 min using a Heraeus Megafuge 16R centrifuge (Thermo Scientific) with a fixed-angle rotor (radius: 8.3 cm, model: F15-6x100, Thermo Fisher). After centrifugation, the supernatants were carefully transferred to new sterile tubes without disturbing the pellet. Finally, the clarified supernatants were stored at -80 °C until further titration.

### IAV titration by plaque assay

A monolayer of MDCK cells was formed in a 6-well, clear, flat-bottom, tissue culture-treated plate (CLS3516, Corning). The culture medium was removed, and the wells were washed twice with 1x PBS pH 7.4. Subsequently, 500 µL of the corresponding viral dilution in DMEM supplemented with Trypsin-TPCK [2 μg/mL TPCK (T1426, Sigma-Aldrich), Trypsin-EDTA (0.02%-0.1 mM, T2601, Sigma-Aldrich)] was added to each well, leaving one well with only DMEM as an uninfected control (mock). The plate was incubated for 1 h at 37 °C in a 5% CO_2_ atmosphere.

*NOTE:* Serial 1:10 dilutions of the viral supernatant to be titrated were prepared beforehand.
*RECOMMENDATION* Scale the volume according to the well size, ensuring that the medium is just sufficient to cover the cell monolayer.
*RECOMMENDATION* To avoid underestimating the viral titer, incubation conditions should be adapted to the virus under study and its recommended propagation conditions.

After the incubation period, the infection medium was carefully removed without damaging the monolayer, and 3 mL of semi-solid media [50% v/w 2x DMEM - 4% methylcellulose (M0512, Sigma-Aldrich)] was added to cover the monolayer (5 mm thickness). The plate was incubated under the previously described conditions for 48 h [27]. Without removing the semi-solid medium, 2 mL of violet fixative (0.25 g crystal violet, 40 mL H_2_O, 10 mL methanol) was added to each well, and the plate was incubated for 30 min at 4° C. The stained semi-solid medium was removed, taking care not to damage the monolayer, then washed with running water, and allowed to dry. The well with the dilution that allowed counting of the plaques was identified, and the viral titer was calculated in plaque-forming units per milliliter (PFU/mL) using **Equation** 2.


$$\frac{PFU}{mL}=\frac{Number\;of\;plaques\;counted}{\left(dilution\;of\;counted\;well)(volume\;of\;infection\right)}$$ (2)

**Equation 2. Calculation of PFU/mL.** The determination of the number of lytic plaque-forming units per milliliter is made from the plaque count obtained experimentally in relation to the count dilution and the sample volume used to infect the monolayer.

### IAV titration by dynamic light scattering

Viral particle quantification by DLS is based on the proportional relationship between the amount of suspended particles and the changes in light deviation caused by their movements. Viral particle quantification was performed using a simple method on a Litesizer DLS 500 instrument with Kalliope software version 2.22.2, both from Anton Paar. The protocol described in this peer-reviewed article is published on protocols.io, doi:dx.doi.org/10.17504/protocols.io.8epv52wr4v1b/v1.

### IAV titration by TCID_50_

In a 96-well, clear, flat-bottom, tissue culture-treated plate (CLS3599, Corning), 1x10^5^ cells/well of the MDCK cell line were seeded with 180 µL of DMEM supplemented with 5.5% FBS. Cells were added to all wells except those in column 10, used to separate infected wells (columns 1–9) from uninfected wells (controls, columns 11 and 12). The plate was incubated at 37° C in a 5% CO_2_ atmosphere. After 24 h, the plate was infected as follows: 20 µL of each viral supernatant was added to the first well of each odd-numbered row (i.e., wells 1A, 1C, 1E, and 1G), and a duplicate was made in the following row (i.e., 1B, 1D, 1F, and 1H). Subsequently, serial dilutions (1:10 dilution, transferring 20 µL from one well to the 180 µL of the next well) were made across the plate from column 1–9. Column 10 remained empty, and columns 11 and 12 were supplemented with supernatant from the uninfected culture (mock controls). Each well was observed daily for cytopathic effects, and the dilution without these effects were recorded. These data were analyzed by the Reed and Muench [2] method using **Equation** 3.


$$\frac{\log\left[TCID_{50}\right]}{mL}=\log\left[{dA}_{50}\right]+\left[\frac{vA_{50}-0.5}{vA_{50}-vB_{50}}\right]+\log\left[\frac{1}{V_{I}}\right]$$ (3)

Equation 3. Calculation of TCID50/mL. This determination is made from the relationship between the dilutions of the wells positive and negative for viral infection and the sample volume used to infect said well. A_50_ is the value above the point where 50% of the wells showed cytopathic effects, and B_50_ is the dilution below that point. d is dilution, v is value, and V_I_ is the inoculum volume in mL.

### Data analysis

A statistical analysis was conducted to evaluate the relationship between the standard curve and cross-method extrapolation data. Pearson’s correlation coefficient was calculated to assess the strength of the linear relationship, and a two-tailed p-value was used to determine statistical significance. Additionally, simple linear regression analysis was performed to obtain the best-fit line and associated 95% confidence limits. All analyzes were performed in the Prism software (v10.1.1) from GraphPad Software, Inc.

## Expected results and discussion

In this study, we developed and validated a viral quantification technique using dynamic light scattering (DLS), which shows a high correlation with traditional viral titration methods such as plaque assays and TCID_50_. This technique offers several advantages over conventional methods, including its speed, independence from cell viability, and ability to measure intact viral particles.

### Influenza A particles meet the conditions to be efficiently characterized by DLS

Since DLS assumes that particles are spherical when calculating size distribution, viral morphology plays a crucial role in obtaining accurate readings. Influenza A viral particles can exhibit pleomorphism in cell culture, presenting as spherical, filamentous, or irregularly shaped forms depending on the strain, host cell type, culture conditions and time post-infection of samples collection. It has been demonstrated that, for IAV PR8, for which this protocol is established, virions produced in MDCK cell culture are predominantly spherical, symmetrical, and range in diameter from 80 to 120 nm [15]. However, to eliminate the low possibility of particles with large, irregular shapes, multimerizations or agglomerations, clarifications can be performed as described in the methodology section.

### There is a high correlation between the values of viral titers by plaque assay and the absolute intensity of scattered light

The plaque assay is the gold standard for IAV titration [26], measuring the formation of viral plaques in cell cultures. This method, while accurate, is labor-intensive and time-consuming, as it requires several days (or even one week for IAV) to obtain results [5,21]. Additionally, it depends on the virus ability to form plaques, which can be a limitation in some cases. In contrast, the DLS titration technique developed in this study allows quantification in a significantly shorter time (1–2 h) and does not rely on plaque formation, offering a more efficient alternative.

**Fig 2:**
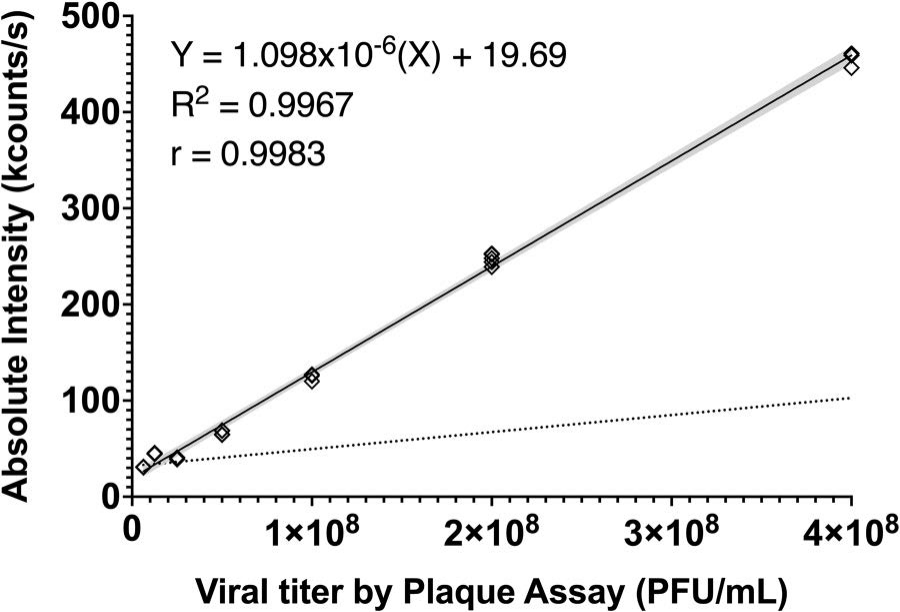
Calibration curve by DLS for IAV titers from MDCK culture. The absolute intensity of light scattered by DLS exhibited a directly proportional relationship with the PFU/mL of Influenza A PR8 virus determined by the plaque assay. The linear regression equation obtained was subsequently used to determine equivalent viral titers of unknown samples. Dilutions were monitored and adjusted as needed to ensure that readings remained within the established methodological parameters. The gray dotted line represents the scattered light intensity limit of the mock supernatant, which did not cause significant interference with the viral supernatant readings. The data presented are based on 26 readings from different serial dilutions of previously titrated supernatants. The shaded area represents the 95% confidence interval.

To validate the viral titration protocol using DLS, clarified supernatants, previously titrated by plaque assay (4.4x10^8^ PFU/mL) were serially diluted and subjected to DLS analysis. Additionally, the clarified supernatant from an uninfected cell culture (mock) was analyzed to exclude potential interference from cellular debris. As shown in **Fig 2**, a high correlation (r = 0.9983, p < 0.0001) was found between the number of plaque forming units per milliliter (PFU/mL) and the absolute intensity of scattered light (kcounts/s), also demonstrating a strong linear relationship (R^2^ = 0.9967, p < 0.001), suggesting that DLS could be a viable and faster alternative for viral particle quantification. Furthermore, it is noteworthy that the scattered light intensity from the mock supernatant did not exhibit a signal that produced significant interference with the readings of the viral supernatants (Absolute Intensity<20 kcounts/s). These results are consistent with previous studies that have compared optical techniques with conventional viral titration methods [5,12,17,23–25,28–30].

**Fig 3:**
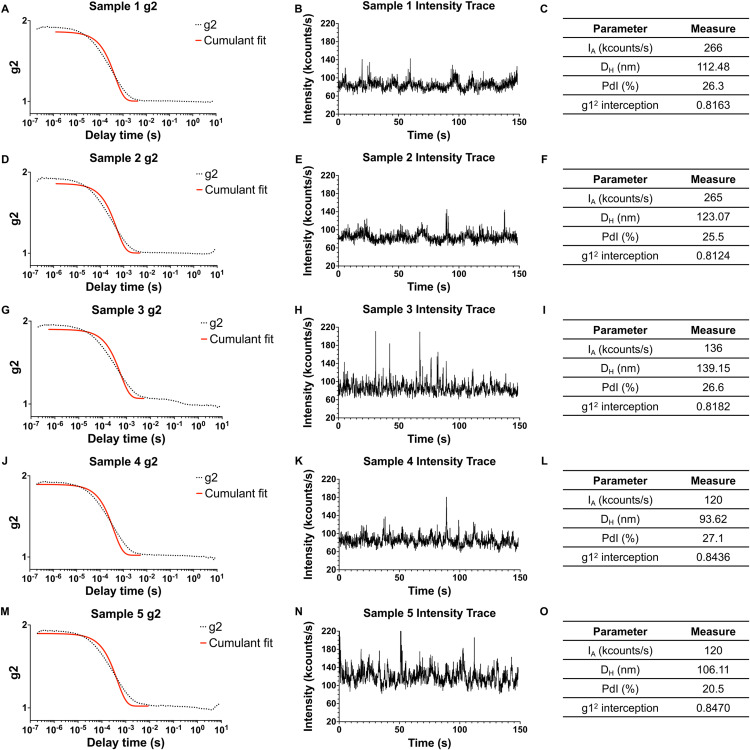
Quality Control Parameters for DLS Viral Supernatant Readings. The figure displays quality control metrics from Dynamic Light Scattering (DLS) readings of five selected viral supernatant samples. Each sample’s data is organized by row, showcasing (A, D, G, J, M) the correlation function fit, (B, E, H, K, N) intensity trace profile, and (C, F, I, L, O) key quantitative parameters, including absolute intensity (IA), hydrodynamic diameter (DH), polydispersity index (PdI), and the g1^2^ intercept. This arrangement allows for a comparative assessment of sample consistency and reading reliability across multiple quality control parameters.

As illustrated in **Fig 2**, the maximum viral titer determined was 4x10^8^ PFU/mL, which remained within the recommended scattered light intensity limit of 600 kcounts/s. As previously discussed, adherence to this threshold is crucial for preventing detector saturation, correlation function errors, instrument instability, and multiple scattering artifacts, all of which can compromise data accuracy. Rigorous monitoring of control parameters, including expected hydrodynamic diameter, polydispersity index, intensity trace profile, and correlation function fit, ensured data quality. While some degree of variability is expected due to the biological origin of the samples, a certain level of flexibility in measurements was allowed, provided that a strong correlation with a standard titration method was maintained. The specific flexibility allowed for each parameter is detailed in the protocol associated with this publication, ensuring that all measurements remained within acceptable ranges while maintaining reliable particle size estimation. All measurements remained within acceptable ranges, ensuring reliable particle size estimation (**Fig 3**).

### Measurements of IAV supernatants with unknown viral titers showed good correlation with TCID_50_, another standardized titration method

After establishing the measurement parameters and constructing the standard curve (**Fig 2**), clarified supernatants from different cell cultures were measured. Dilutions were performed based on the monitoring of the absolute intensity of scattered light. Finally, using the linear regression equation (*y = 1.098 × 10*^*-6*^ *x + 19.69*), equivalent titers in terms of PFU/mL (eqPFU/mL) were calculated (**Equation** 4). Some representative data of this determination are shown in **Table 1**.

**Table 1:** DLS-based titrations of unknown samples.

Read	Dilution factor	I_A_ (kcounts/s)	eqV_T_ (eqPFU/mL)
1	1^*^	301	2.56x10^8^
2	1^*^	68	4.40x10^7^
3	2	441	7.67x10^8^
4	2	261	4.40x10^8^
5	20	109	1.63x10^9^
6	20	159	2.54x10^9^
7	30	211	5.23x10^9^
8	30	220	5.47x10^9^

I_A_: Absolute Intensity; eqVT: equivalent viral titer; eqPFU/mL: equivalents to plaque forming units per mL.

* Undiluted sample, but factor is considered as 1 for use in the formula described.


$$eqV_{T}=\frac{I_{A}-19.69}{1.098\times 10^{-6}}\times dilution$$ (4)

**Equation 4. Formula for equivalent titers by DLS.** For the calculation, we considered the absolute intensity of each sample’s readings, the experimentally established relationship factor between intensity and viral titer under our specific conditions, and adjustments for the dilution applied.

**Fig 4:**
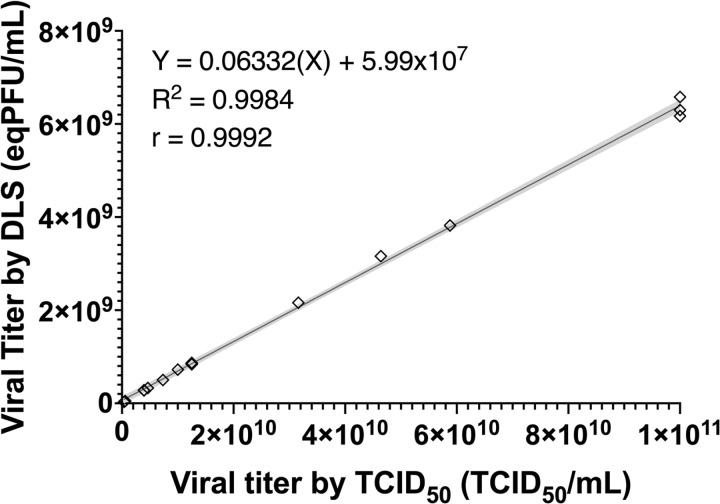
Correlation between IAV titers determined by TCID_50_ and DLS. The equivalents titers by DLS exhibited a directly proportional relationship with the TCID_50_/mL of IAV titers. Dilutions were monitored and adjusted as needed to ensure that readings remained within the established methodological parameters. The data presented are based on 16 readings from different supernatants. The shaded area represents the 95% confidence interval.

To validate the equivalent titers obtained, another highly accepted titration method for IAV, the TCID_50_ method, based on determining the dose of virus required to cause a cytopathic effect in 50% of infected cells, was employed. Although this method is also a standard in virology, it presents similar limitations to the plaque assay, including variability in cell culture conditions and the time required to obtain results. As shown in **Fig 4**, a high correlation (r = 0.9992, p < 0.001) and a good linear fit (R^2^ = 0.9984, p < 0.001) were found between the titers determined by TCID_50_ and DLS (on terms of eqPFU/mL). The high correlation observed between DLS and TCID_50_ obtained reinforces the utility of DLS as a complementary or even alternative technique for viral quantification. Previous studies have shown that methods such as TCID_50_ can be sensitive to cell culture conditions [5,18], which could limit their reproducibility compared to light-scattering-based techniques.

In addition to traditional methods, viral quantification is also commonly performed using molecular techniques such as qPCR, which measures the amount of viral nucleic acid and is extremely sensitive, allowing the detection of viral genomes even at low concentrations [17,26]. However, a key limitation is that qPCR does not provide information about viral infectivity, as it does not measure the presence of complete and infectious viral particles [3,5]. In contrast, DLS measures light scattering from viral particles, which may offer an advantage in detecting the overall particle population, including potentially incomplete non-infectious virions, as it does not provide information about viral genome packaging. However, DLS allows a rapid and reproducible relationship of total viral particle production.

Another method is Nanoparticle Tracking Analysis (NTA), which measures the size and concentration of particles in solution by tracking the movement of individual particles [31]. Although NTA and DLS are complementary techniques, DLS offers an advantage in terms of speed, reproducibility and simplicity, as it does not require direct visualization of individual particles and may be more suitable for different samples with a broad viral particles concentrations (10^8^ to 10^12^ particles/mL for DLS vs 10^7^ to 10^9^ particles/mL for NTA) [28,32]. The use of NTA may be preferable in cases where resolving individual particle populations is required.

As far as we know, other DLS-based protocols for viral titer determinations have failed to show such a good correlation as this study [23,24,29,30], requires more specialized equipment or adaptations with a smaller range of applications [33], have a high influence of background noise [22], or an expensive, time-consuming, denaturing or inadequate sample handling procedures [25,32], leading to poor sample preservation, imprecise readings and consequently low reproducibility, precision, and accuracy.

A key benefit of the presented protocol is its label-free nature, eliminating the need for additional sample treatments beyond centrifugation. This simplifies the workflow, reduces costs, and avoids the complexities associated with viral particle labeling and the requirement for specific markers for each viral strain. While PA and TCID_50_ gives a direct count of infective virus particles, non-infective virus particles do not produce plaques or cytopathic effects, and aggregates containing many virus particles will produce only single plaques. Often, the only need is to know the number of virus particles, whether infective or not. Finally, another possible application is to monitor the integrity of the quality of viral particles after preservation processes, such as freezing, for long periods.

Limitations of this technique include the inability to determine titers greater than approximately 5x10^9^ PFU/mL (based on our experience with IAV), necessitating dilutions that limit sample recovery. Conversely, for lower initial viral concentrations, larger sample volumes are required, demanding at least 1x10^7^ PFU/mL titers. In case of having very low titers, Driskell et al. demonstrated the rapid quantification of IAV using DLS, achieving high sensitivity (<100 TCID_50_/mL) through the use of antibody-conjugated gold nanoparticles [25]. Finally, it is important to note that while DLS titration is based on infectious particles, interpolation for unknown samples only relates to complete particles and not infectivity. Although this may be sufficient for assays such as antigenicity, vaccine development, or antiviral response assays, if infectivity is required, this protocol can serve as a preliminary screening tool, with infectivity confirmed by classical assays.

The adaptation of this protocol for viruses other than IAV should be straightforward and feasible. While this study primarily focused on the enveloped influenza A virus, the methodology presented here is expected to be adaptable to non-enveloped viruses. The absence of a lipid envelope in naked viruses may result in even less variability in particle size and polydispersity, making DLS measurements potentially more straightforward. Future studies could explore the application of this protocol to a broader range of viruses, including those with icosahedral or filamentous capsids, to further assess the versatility and limitations of DLS in viral particle characterization. It is important to highlight that, as shown previously, standardization of the analysis can be initiated from PFU or TCID_50_, the two most commonly used methods for cytopathic viruses.

## Conclusion

DLS emerged as a rapid and reliable method for quantifying viral particles in this study. By adhering to rigorous experimental protocols, including the maintenance of optimal intensity levels, we demonstrated the accuracy and precision of this technique with non-destructive measurements and no or minimal sample preparation, in minutes. The strong correlation between DLS-derived viral titers and those obtained from traditional gold-standard methods, such as plaque assay and TCID50, validates its utility as a robust quantification tool.

While DLS excels in providing rapid and quantitative assessments of viral particle concentration, it is essential to acknowledge its limitations. The inability to distinguish between infectious and non-infectious particles necessitates the use of complementary techniques for a comprehensive understanding of viral infectivity. Nonetheless, DLS offers a valuable initial screening tool to prioritize samples for further characterization.

The potential applications of DLS extend beyond basic viral quantification. Its speed and sensitivity make it a promising tool for monitoring viral load in clinical settings, evaluating vaccine efficacy, and assessing the potency of viral-based therapeutics. As DLS technology continues to advance, we anticipate its integration into routine virological workflows, contributing to accelerated vaccine development, improved disease surveillance, and enhanced patient care.
